# Influence of abiotic factors on diapause termination and temperature requirements for postdiapause development in the European red mite, *Panonychus ulmi* (Acari: Tetranychidae)

**DOI:** 10.1007/s10493-024-00904-9

**Published:** 2024-04-18

**Authors:** E. Martínez-Villar, B. López-Manzanares, S. Legarrea, I. Pérez-Moreno, V. S. Marco-Mancebón

**Affiliations:** 1 Departamento de Agricultura y Alimentación, Universidad de La Rioja, Madre de Dios 53, 26006 Logroño, La Rioja, Spain; 2 Departamento de Viticultura, Instituto de Ciencias de la Vid y del Vino (ICVV), Finca La Grajera, Carretera de Burgos, km 6, 26071 Logroño, La Rioja, España

**Keywords:** Diapause, Mite, Modelling, Abiotic conditions, IPM

## Abstract

The European red mite *Panonychus ulmi* (Koch) is widely distributed and it can severely affect pome fruit crops, particularly apple. Pest outbreaks are related to an overuse of non-selective pesticide treatments that lead to the development of resistance and the absence of natural enemies in the orchard. A key aspect to optimize the use of pesticide treatments in the context of IPM is to increase the knowledge on the biology and ecology of the pest to better predict population dynamics and outbreaks. For the European red mite, knowledge on the conditions that lead to diapause breaking by winter eggs is essential to model population dynamics. To increase this knowledge, winter eggs were collected during field surveys in northen Spain during three years and egg hatching was monitored under controlled temperature and photoperiod conditions in the laboratory. The “number of days exposed to cold temperatures” was the most significant factor that positively affected hatching of overwintering eggs. The time required for 50% of the egg population to hatch (T_50%_) was also negatively modulated by the duration of exposure to cold temperature. The temperature threshold for postdiapause eggs development collected from the field was estimated between 5 and 6 ºC in 2005 and 2007, respectively. Moreover, the degree-days required for post diapause development were estimated between 263.2 and 270.3, depending on the year of collection. Collectively, we provide additional information on the diapause termination and postdiapause development of the European red mite that may effectively contribute to optimize pest population models.

## Introduction

*Panonychus ulmi* (Koch) (Acari: Tetranychidae) is considered one of the most important mite pest affecting deciduous fruit crops, and it is currently present in about 71 countries, distributed in Europe and other continents (Bolland et al. 1998; Migeon and Dorkeld 2023). Not only is *P. ulmi* widespread, but it is also polyphagous, reported on 151 host plants belonging to more than 37 families (Migeon and Dorkeld 2023). The family Rosaceae contains the largest number of species attacked by *P. ulmi* (Migeon and Dorkeld 2023), with a major impact on apple (*Malus domestica* Borkh.) cultivation worldwide (Costa-Comelles et al. 1991; García de Otazo 1992; García-Marí et al. 1994). As with other tetranychiid mites, damage develops from feeding by the mobile forms on the leaves, causing a reduction of leaf chlorophyll that may lead to a significant yield reduction. In other hosts like pear (*Pyrus communis* L.), tolerance to mite feeding is very low and few individuals may cause serious leaf burning (Hoyt and Tanigoshi 1983). In severe infestations, mites can colonize fruits, leading to a reduction in size and loss of color (Botha and Learmonth 2005). Additionally, *P. ulmi* infestations may cause changes to hormone and mineral levels that could reduce floral induction in the following year (Tomczyk and Kropczynska 1985).

Despite the importance of this mite pest, research on the biology and ecology of *P. ulmi* is still limited, particularly when compared to other mite species such as *Tetranychus urticae* Koch. A search in Scopus database showed that the number of publications on *T. urticae* is ten times higher than *P. ulmi* (4251 versus 485, respectively; search conducted on the 19th Feb 2024). Furthermore, only 20 of the 485 documents on *P. ulmi* focused on diapause (Scopus search: (TITLE-ABS-KEY(panonychus AND ulmi) AND TITLE-ABS-KEY(diapause*))). This analysis emphasizes the need for additional studies on a worldwide pest such as *P. ulmi*. Among the factors that may limit conducting research on *P. ulmi* are the difficulties that entail rearing this mite species under laboratory conditions. For example, *P. ulmi* is generally found and reared on a woody host plant with obvious challenges of space to accommodate all required materials. Alternatively, instead of establishing laboratory rearings, individuals may be collected from the field prior to the experiments. However, variable climatic conditions experienced by field populations may influence mite performance. Specifically, winter diapause may drastically affect hatching success in the laboratory. Thus, increasing the knowledge of the conditions under which *P. ulmi* overcomes diapause can be applied to optimize methodologies and facilitate research on this species.

Diapause is a biological phenomenon commonly deployed by arthropods to survive periods of difficult environmental conditions that may compromise their survival (Denlinger 2002). It is defined as a genetically determined state in which development is suppressed and controlled by environmental factors (Bale and Hayward 2010). A quiescent stage allows these arthropods to synchronize their biological cycles with the phenology of their host plant and also, to establish their pattern of voltinism, that is, the number of cycles that the species complete per natural year (Veerman 1985). Diapause has been investigated for several mite groups, including tetranychiids (Vacante 2016), among which the presence of adult female and dormant eggs are common (Veerman 1985). For *P. ulmi*, diapause occurs at the stage of eggs during the winter period, a process of dormancy denoted as hibernation (Saunders 1982). In our area of study, located in northern hemisphere, in the Mediterranean region (42º24’38.83’’N; 2º26’2.13’’W), the physiological process of diapause starts in middle August. Photoperiod, temperature and, to a lower extent, the availability of food are three factors that signal females to lay “winter eggs”, those that will experience diapause (Van de Vrie 1985). The period of laying winter eggs may extend from August to October and even, late November, depending on the environmental conditions (Van de Vrie 1985). It was estimated that females that lay winter eggs appear upon a light photoperiod of 6 to 13 h and an average temperature around 15 ºC (Lees 1953). Once winter eggs are laid, they develop until the stage of blastoderm, at which the mite experiences diapause (Gotoh et al. 1994).

Diapause termination occurs once the environment is favorable for mite’s development. The main factors that determine the optimum environmental conditions are temperature, and the period of time that an organism is exposed to it (Lees 1953; Light et al. 1968; Cranham 1971, 1972, 1973; García-Marí et al. 1991; Evans 1992; Koveos and Broufas 1999). Most research on the effect of photoperiod was devoted to understand the conditions that lead females to lay winter eggs (Lees 1953), while the role of this environmental factor on diapause breaking has been less explored. Lees (1953) observed that egg hatching of *P. ulmi* could be documented at complete darkness under laboratory conditions (Table 12 in Lees 1953), thereby, attributing a minor role of photoperiod on egg development. However, photoperiod can control the induction and breaking of diapause for other tetranychiid mites such *T. kanzawai* Kishida (Shah et al. 2011) and *T*. *urticae* (Koveos and Veerman 1996). For this species, the effect of photoperiod can be qualitative and quantitative (Kroon et al. 1998). Nevertheless, *T. urticae* differs from *P. ulmi* in that diapause occurs at different stages, therefore it is still unclear how similar the impact photoperiod on diapause termination will be for both species.

In the light of a changing climate, it is necessary to study mite diapause and postdiapause development, not only to better understand mite biology, but also to adjust models and predict population dynamics and its range of biological expansion. *P. ulmi* requires a period of cold temperature to complete diapause and resume the cycle in spring. Determining the minimum period of cold days required to break diapause is highly relevant to predict the moment of emergence of the population in the following season. For example, it was observed that an uneven diapause breaking may lead to overlapping summer populations for several species and this may compromise pest management strategies. For the case of *P. ulmi*, degree-day models predicted that the number of generations for *P. ulmi* in California under a climate change scenario would dramatically increase by at least 40% in walnut crops (from 9 to 14 generations to 14–20 generations per year) (Luedeling et al. 2011). However, further information relevant to this mite species (such as the specific conditions that influence diapause) could be integrated in the modelling approaches, to improve the prediction of yearly population dynamics.

In this work, the aim was to determine the influence of temperature, duration of exposure to cold, and photoperiod on diapause breaking of *P. ulmi*. For that purpose, field surveys to collect winter eggs were conducted during three years. Furthermore, batches of eggs were exposed to several environmental conditions by manipulating temperature and photoperiod under laboratory conditions and observed until egg hatching. The percent of hatched eggs and the time required for 50% of the population to hatch were determined as the two main parameters that are modulated by abiotic factors. Additionally, relevant parameters such as the degree-days and the temperature threshold required for postdiapause development were estimated. The application of this work can be two pronged. First, we increase the knowledge of the conditions required for egg hatching, which can contribute to the optimization of laboratory experimental methods. Second, our data can be used to customize models on mite development and distribution of expansion range in the context of climate change.

## Materials and methods

### Field sampling and species of study

Winter eggs of *P. ulmi* were collected on apple (var. Reineta Blanca de Canadá) twigs in an orchard located in Alberite (La Rioja, Spain, 42º24’38.83’’N; 2º26’2.13’’W) at an altitude of 449 m. Weather data was obtained from a nearby agroclimatic station (Logroño, SIAR, 42º26’22.88’’N; 2º30’49.09’’W, altitude: 465 m) and it is presented as supplementary information for the years of study (SI). Tree phenology was estimated using the scale determined by Fleckinger (1945) in an apple orchard in Logroño. Following this approach, the date of full bloom (F2 stage: anthesis of 50% of the flowers) was established as the 16th of April in 2007. Overall, apple full bloom occurs in middle April in our area of study.

Surveys to collect egg masses were conducted in autumn season (month of November) during three years (2004, 2005 and 2007). Winter eggs were distinguished by size, color, and the location of the egg masses. Compared to summer eggs, winter eggs are larger and darker red in color (García de Otazo 1992). Winter eggs are mainly deposited on the bark of the branches that are at least 2 years old, around the floral buds and in areas that may provide refuge, such as the insertion of branches to the main trunk or small crevices; with higher frequency on the side of the tree facing south.

### Experimental units

Experimental units consisted of small portions of bark containing groups of 30 to 100 winter eggs. Each portion of bark was fixed with glue (Pritt Roller®) to a filter paper inside a Petri-dish (9 cm diameter). To avoid the escape of emerging mites, a circular layer of glue or Vaseline® was deployed to enclose the arena.

### Bioassays to study the factors that influence diapause termination

To unravel which environmental conditions (temperature, duration of exposure to cold and photoperiod) may affect diapause termination, winter eggs were exposed subsequently to two environmental conditions. First, egg masses were exposed to a variable period of cold temperatures and complete darkness. In particular, four temperatures were assayed (0, 2, 4 and 8 ºC) during different time periods (10, 20, 40, 60, 80 and 100 days). Subsequently, half of the eggs from each treatment were exposed to conditions of long photoperiod (16:8 h L:D) and the other half to a short photoperiod (8:16 h L:D), being all experimental units maintained at 20 ºC. Altogether, the experimental approach comprised 48 treatments; the factorial combination of four experimental temperatures, six periods of time and two photoperiod conditions. For each treatment, three to four replicates were included. The experiment was conducted during two consecutive seasons and data was analyzed separately for each year, giving the possible effect of meteorological conditions on the winter eggs at the start of the experiment. Egg hatching was evaluated every 10 days by counting the number of larva emerged from the eggs. The evaluation period was finalized for each experimental arena if no emerging mites were observed after two consecutive observations (i.e. 20 days). This dataset served to estimate the percentage of egg hatching success as well as the time required for half of the mite population to hatch (T_50%_).

### Bioassay to determine the starting date of postdiapause

Eggs collected from the field surveys were exposed to environmental conditions similar to the open field, but protected from rainfall, on a covered terrace located at the University of La Rioja (Logroño, La Rioja, Spain, 42º28’0.91’’N; 2º25’22.51’’W). From January onwards egg masses of at least 50 eggs were brought to the laboratory every two to three days and set up in experimental arenas as above. Two replicates were set up per each day of evaluation. Experimental arenas were exposed to controlled environmental conditions (20 ºC; 16:8 h L: D photoperiod; 60–70%RH) and checked for egg hatching at a frequency of three to four days for a total period of 60 days. Diapause termination was determined on the date at which 50% of the eggs in the population survey had hatched (Koveos and Broufas 1999).

### Bioassay to determine the temperature threshold and degree-days required for postdiapause completion

These assays were performed with egg masses collected during two years. In 2005, egg masses maintained in outside conditions (as described in 2.4) were collected every 2–3 days starting on the 18th January until the 21th February. In 2007, eggs were collected from the 17th of January until the 19th of March. For each test, two egg masses of approximately 50 eggs were exposed to constant temperatures (4, 9, 14 and 19 ºC) at total darkness and 60–70% RH. Every 2–3 days, the number of eggs hatching was evaluated. Data obtained starting from the date established as the “starting day of postdiapause in Sect. 2.4” onwards were used to calculate the number of days required for egg hatching after diapause termination. The inverse value of this number of days (1/t) was established as the “egg developmental rate for postdiapause”. The graphical representation of this rate allowed to calculate the “threshold temperature for postdiapause development” as the temperature at which the regression equation crosses the x-axis (y-axis = 0). The degree-days required for postdiapause development were calculated as the reciprocal of the slope of the regression equation (Campbell et al. 1974; Broufas and Koveos 2000). Climatic data obtained by a nearby agroclimatic station (Logroño; SIAR: https://www.larioja.org/agricultura/en/informacion-agroclimatica/red-estaciones-agroclimaticas-siar) was used to predict the date of 50% egg-hatching based on the estimation of the threshold temperature and the degree-days required for postdiapause development.

### Statistical analysis

Statistical analysis was performed using SPSS v. 10.0. (SPSS Inc., 1999). The average hatching percentage of mites per experimental unit was analyzed using a full factorial three way ANOVA, including temperature, duration of cold temperature and photoperiod as fixed factors. Multiple comparisons to test for significant differences were performed using post-hoc tests (Tukey HSD, α = 0.05). Additionally, the number of days required for 50% of the eggs to hatch, considering only those that successfully hatch over the entire period was selected as a parameter that estimated diapause breaking (Koveos and Broufas 1999) and the statistical analysis was performed as described above for this parameter.

## Results

### Factors affecting postdiapause egg hatching

**Fig. 1:**
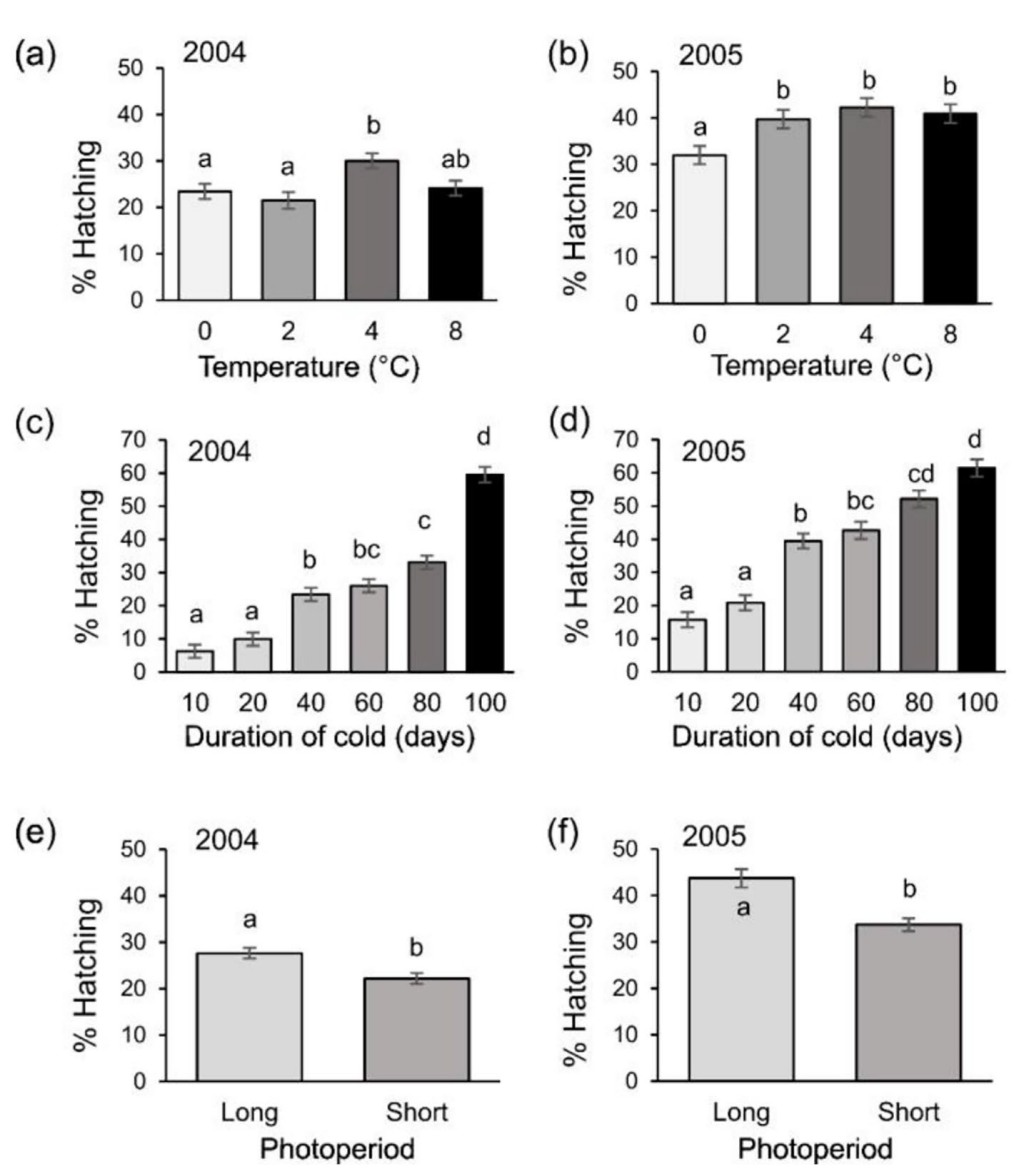
Egg hatching success (mean ± SE) influenced by (**a**, **b**) temperature, (**c**, **d**) duration of cold temperature and (**e**, **f**) photoperiod for the European red mite incubated at 20 ºC in two years of study: 2004 (panels **a**, **c**, **e**) and 2005 (panels **b**, **d**, **f**). Different letters indicate significant differences after ANOVA followed by Tukey post-hoc test (α = 0.05)

The analysis of all factor combinations showed that for both years, the final percentage of egg hatching was influenced by the three factors under study: temperature, period of cold and photoperiod (Table 1; Fig. 1). Significant interactions were only detected for the factors “cold duration” and photoperiod in 2005. In this season, increasing the duration of cold temperatures was generally affecting the percentage of hatching when eggs were exposed to a short photoperiod. In contrast, in 2004, the percentage of hatching increased with the duration of cold temperatures at both conditions of long and short photoperiod.

**Table 1:** Statistical output of the three-way ANOVA on the percentage of European red mite eggs hatching after exposure to different temperatures, duration of cold temperature and photoperiod under laboratory conditions. Statistically significant P-values are highlighted in bold

Variables		Year 2004				Year 2005		
		df	F	*P*		df	F	*P*
Photoperiod (P)		1, 138	9.95	**0.002**		1, 119	25.13	**0.000**
Cold duration (D)		5, 138	77.05	**0.000**		5, 119	53.16	**0.000**
Temperature (temp)		3, 138	3.75	**0.012**		3, 119	5.40	**0.002**
P*D		5, 138	2.04	0.077		5, 119	5.11	**0.000**
P*temp		3, 138	0.75	0.523		3, 119	1.41	0.245
D*temp		14, 138	1.25	0.250		15, 119	1.64	0.072
P*D*temp		14, 138	1.01	0.450		15, 119	0.75	0.718

Overall, the period of time at which eggs experienced low temperatures influenced egg hatching; that is, the longer the period of exposure to cold, the higher the percentage of egg hatching success. Figure 1 shows that egg hatching was the highest at 60% when the cold temperatures were applied consecutively for 100 days. Intermediate values of egg hatching were obtained for periods ranging from 40 to 80 days of cold temperature, while less than 30% of the eggs hatched if less than 20 days of cold temperature were experienced. Additionally, the photoperiod at which eggs were exposed upon termination of the cold treatment was a significant factor modulating diapause breaking. Exposure to a long photoperiod significantly increased the percentage of eggs that hatched (Fig. 1). Particularly, a long photoperiod significantly increased egg hatching when the number of cold days was short (10, 20, and 40 days) (Table 2). Finally, temperature alone had a significant effect on egg-hatching but this trend was different depending on the year of study. Figure 1 shows that the optimum temperature for increasing egg hatching was 4ºC in 2004. Conversely, in 2005 the overall percentage of egg hatching was higher than 2004; and a range between 2 and 8 ºC was sufficient to increase the percentage of eggs hatched compared to exposure to 0 ºC.

**Table 2:** Percentage of egg hatching (average ± SE) for the European red mite upon exposure to specific conditions of cold temperature, for a determined period of time and subsequently exposed to conditions of long or short photoperiod

Year	Temp. (ºC)	Photoperiod^a^	Duration of exposure to cold temparature (days)					
			10	20	40	60	80	100
2004	0	Long	8.34 ± 2.03 a	7.32 ± 1.72 a*	28.27 ± 5.12a*	24.19 ± 6.73 a	37.92 ± 4.81 b	43.92 ± 8.45 b
		Short	3.88 ± 0.95 ab	1.32 ± 1.32 a*	12.43 ± 3.54 ab*	25.25 ± 2.55 ab	28.91 ± 6.45 b	59.30 ± 10.16 c
	2	Long	7.14 ± 1.66 a	21.71 ± 2.14 ab*	36.56 ± 10.18 b	35.86 ± 4.60 b	29.19 ± 7.15 ab	N/A
		Short	2.88 ± 1.67 a	9.68 ± 2.73 a*	14.14 ± 3.43 ab	25.36 ± 1.64 bc	32.28 ± 4.42 c	N/A
	4	Long	10.60 ± 2.67 a*	18.84 ± 1.45 ab*	21.53 ± 2.91 ab	26.67 ± 4.22 ab	35.49 ± 9.40 b	73.84 ± 4.63 c
		Short	1.76 ± 1.12 a*	3.81 ± 2.62 a*	28.12 ± 6.35 ab	26.21 ± 2.94 ab	40.81 ± 8.84 b	72.70 ± 10.06 b
	8	Long	14.39 ± 1.86 a*	11.76 ± 4.64 a	32.58 ± 6.16 ab	24.89 ± 5.03 ab	29.42 ± 9.60 ab	55.05 ± 10.80 b
		Short	1.04 ± 1.04 a*	5.12 ± 1.74 ab	13.53 ± 2.62 ab	19.45 ± 2.90 ab	30.43 ± 7.41 bc	52.05 ± 11.69 c
2005	0	Long	15.41 ± 1.99 a	26.42 ± 3.18 ab*	42.72 ± 7.21 bc	33.38 ± 3.95 abc	58.44 ± 3.69 c*	49.10 ± 8.57 bc
		Short	7.44 ± 3.00 a	8.19 ± 2.70 a*	24.40 ± 11.39 a	29.55 ± 5.84 ab	31.18 ± 5.56 ab*	57.15 ± 3.17 b
	2	Long	28.03 ± 2.63 a*	32.82 ± 3.48 ab*	51.42 ± 17.56 ab	44.14 ± 4.23 ab	52.56 ± 2.56 ab*	72.12 ± 0.43 b
		Short	9.22 ± 2.34 a*	11.82 ± 3.22 ab*	36.04 ± 2.24 ab	39.63 ± 9.90 bc	30.37 ± 4.13 ab*	68.56 ± 13.41 b
	4	Long	27.65 ± 3.91 a*	31.69 ± 8.02 a*	47.33 ± 6.62 ab	36.14 ± 5.33 ab	54.70 ± 4.90 ab	65.83 ± 4.49 b
		Short	11.48 ± 4.40 a*	10.70 ± 3.06 a*	46.56 ± 13.93 ab	54.19 ± 6.07 ab	56.05 ± 8.19 ab	64.85 ± 13.86 b
	8	Long	17.62 ± 4.00 a	34.17 ± 7.72 ab*	42.96 ± 5.45 ab*	47.27 ± 1.37 ab	84.44 ± 6.34 c*	52.58 ± 9.63 bc
		Short	8.53 ± 2.17 a	10.87 ± 3.00 a*	24.10 ± 3.23 a*	57.16 ± 4.04 b	49.33 ± 9.23 b*	61.73 ± 7.30 b

^a^ Photoperiod was established as Long (16:8 h; L:D) or Short (8:16 h; L:D).

N/A indicates treatment combinations for which the number of replicates were not sufficient to estimate the percentage of egg hatching.

Asterisks indicate significant differences in columns between long and short photoperiod for each combination of temperature and days (*t* test: α = 0.05).

Different letters in rows indicate significant differences after one way ANOVA and post-hoc comparisons (Bonferroni, *P* < 0.05).

### Average time to hatch (T_50%_)

**Fig. 2:**
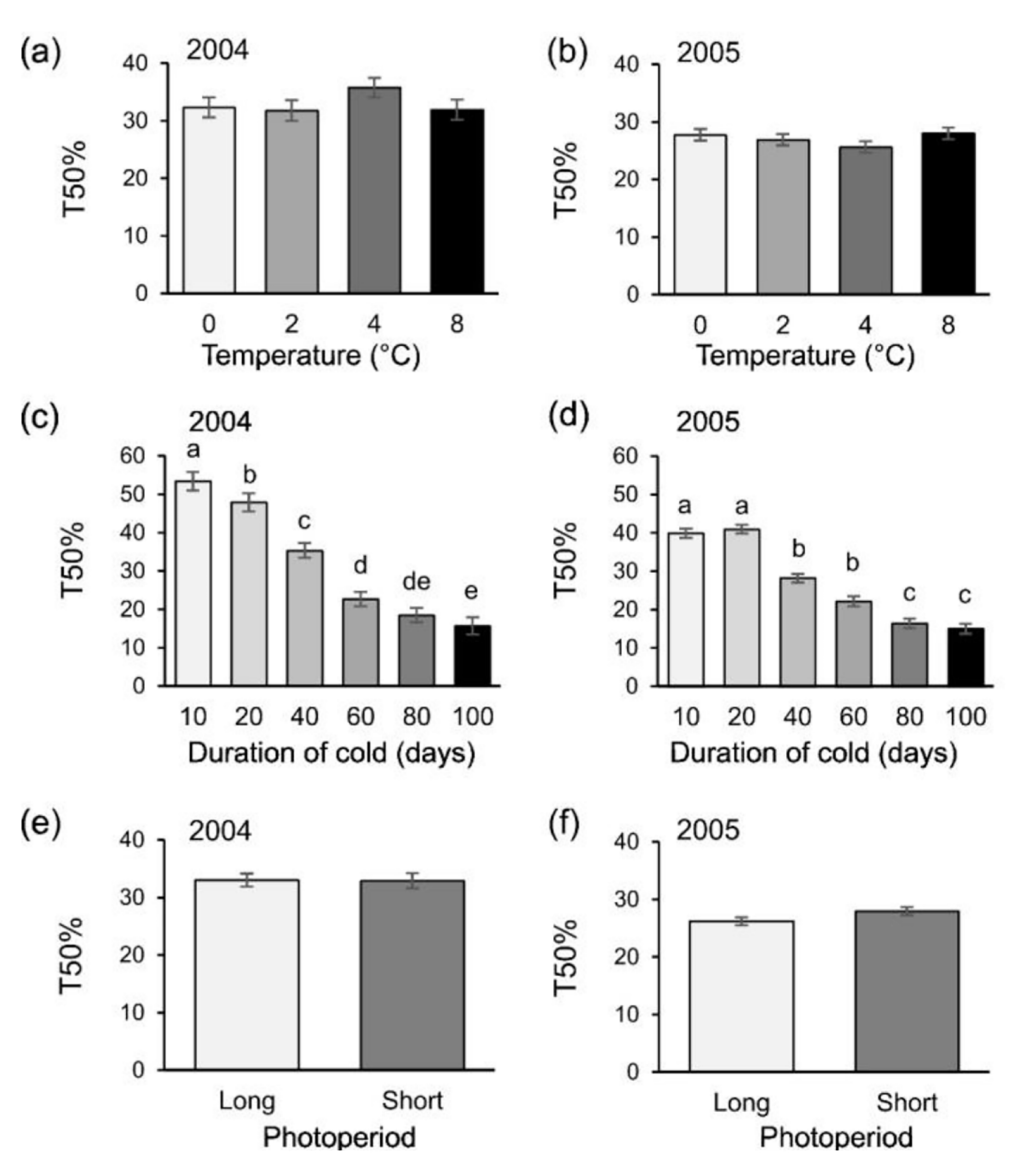
T50% values (mean ± SE) influenced by (**a**, **b**) temperature, (**c**,**d**) duration of cold temperature and (**e**,**f**,) photoperiod for eggs of the European red mite incubated at 20ºC in two years of study: 2004 (panels **a**, **c**, **e**) and 2005 (panels **b**, **d**, **f**). Different letters indicate significant differences after ANOVA followed by Tukey post-hoc test (α = 0.05)

The period of time at which egg masses were exposed to cold temperature significantly influenced the parameter T_50%_ (Tables 3 and 4). In this case, a longer period of exposure to cold temperature decreased T_50%_. At the longest periods of cold (100 − 80 days), T_50%_ was only 15 to 18.5 days. In the opposite scenario, a short period of cold (10–20 days) resulted in a T_50%_ of 41 to 53 days (Table 4). While temperature and photoperiod did not influence T_50%_ alone, these factors modulated the effect of duration of exposure to cold temperature. In particular, significant interactions (*P* < 0.05) occurred between cold duration and temperature during both years, while a three- way intereaction was only significant in 2004 (Table 3; Fig. 2).

**Table 3:** Statistical output of the three-way ANOVA on the T50% required for European red mite eggs to hatch upon exposure under laboratory conditions to different temperatures, duration of cold temperature and photoperiod. Statistically significant P-values are highlighted in bold

Variable		Year 2004				Year 2005		
		df	F	*P*		df	F	*P*
Photoperiod (P)		1, 125	0.014	0.905		1, 117	2.59	0.110
Cold duration (D)		5, 125	45.61	**0.000**		5, 117	98.77	**0.000**
Temperature (temp)		3, 125	1.97	0.122		3, 117	2.55	0.059
P*D		5, 125	5.05	**0.000**		5, 117	0.35	0.882
P*temp		3, 125	1.94	0.126		3, 117	0.32	0.810
D*temp		14, 125	3.45	**0.000**		15, 117	2.93	**0.001**
P*D*temp		14, 125	2.13	**0.014**		15, 117	0.67	0.805

**Table 4:** T50% values (average days ± SE) for the European red mite upon exposure to specific conditions of cold temperature, for a determined period of time and subsequently exposed to conditions of long or short photoperiod

Year	Temp. (ºC)	Photoperiod^a^	Duration of exposure to cold temparature (days)					
			10	20	40	60	80	100
2004	0	Long	73.75 ± 2.39 a	47.5 ± 12.5 b	27.75 ± 1.89 bc*	16.00 ± 1.00 c	14.00 ± 2.00 c	14.00 ± 0.71 c
		Short	57.50 ± 17.02 a	N/A	40.00 ± 2.04 ab*	22.75 ± 2.29 ab	17.5 ± 2.78 b	14.25 ± 0.75 b
	2	Long	44.68 ± 15.13 ab	48.59 ± 2.72 a	35.28 ± 2.61 ab	20.53 ± 0.95 ab	14.99 ± 1.02 b*	N/A
		Short	46.47 ± 14.64 ab	56.42 ± 6.51 a	29.68 ± 3.36 b	24.79 ± 2.14 b	21.94 ± 1.02 b*	N/A
	4	Long	70.33 ± 7.55 a	49.31 ± 3.47 b	29.98 ± 3.40 c	20.13 ± 1.08 c*	16.89 ± 0.58 c*	14.31 ± 0.37 c*
		Short	70.00 ± 15.00 a	53.43 ± 5.23 a	38.61 ± 1.80 b	28.62 ± 1.39 bc*	21.24 ± 1.13 c*	16.52 ± 0.51 c*
	8	Long	57.90 ± 8.75 a	58.72 ± 2.29 a	29.81 ± 7.48 b*	25.99 ± 1.66 b*	19.74 ± 1.57 b	15.49 ± 0.93 b
		Short	N/A	41.49 ± 12.17 ab	53.06 ± 2.93 a*	35.15 ± 1.89 ab*	24.20 ± 0.93 b	17.16 ± 0.72 b
2005	0	Long	44.50 ± 6.60 a	41.75 ± 5.45 ab	24.75 ± 0.48 abc	21.67 ± 0.33 bc*	16.33 ± 4.33 c	14.67 ± 0.33 c
		Short	46.67 ± 1.67 a	39.75 ± 4.40 ab	26.67 ± 1.67 bc	24.33 ± 0.67 c*	14.67 ± 2.67 c	14.67 ± 0.33 c
	2	Long	45.50 ± 3.52 a	35.00 ± 2.04 b	23.25 ± 0.63 c	15.67 ± 1.20 cd*	13.00 ± 0.58 cd	12.67 ± 1.20 d
		Short	41.25 ± 5.15 a	43.75 ± 3.38 a	23.50 ± 1.55 b	20.67 ± 0.67 b*	12.67 ± 0.67 b	13.33 ± 1.20 b
	4	Long	27.75 ± 6.64 ab	32.00 ± 3.39 a*	25.25 ± 0.25 ab	23.33 ± 0.88 ab	16.67 ± 1.20 ab	11.33 ± 0.33 b
		Short	33.00 ± 6.18 ab	42.50 ± 2.50 a*	26.50 ± 2.22 bc	20.67 ± 0.88 bc	19.33 ± 0.88 bc	12.33 ± 1.33 c
	8	Long	31.75 ± 4.68 b	45.75 ± 1.93 a	29.25 ± 1.49 bc	24.00 ± 1.00 bcd	16.33 ± 1.86 cd	10.67 ± 0.33 d
		Short	35.00 ± 4.04 ab	44.50 ± 6.71 a	31.75 ± 0.48 ab	24.67 ± 0.33 bc	16.33 ± 1.33 bc	11.33 ± 0.33 c

^a^ Photoperiod was established as Long (16:8 h; L:D) or Short (8:16 h; L:D)

N/A indicates treatment combinations for which the number of replicates were not sufficient for T_50%_ calculation

Asterisks indicate significant differences in columns between long and short photoperiod for each combination of temperature and days (*t* test: α = 0.05)

Different letters in rows indicate significant differences after one way ANOVA and post-hoc comparisons (Bonferroni, *P* < 0.05)

### Establishing the date for diapause termination

**Fig. 3:**
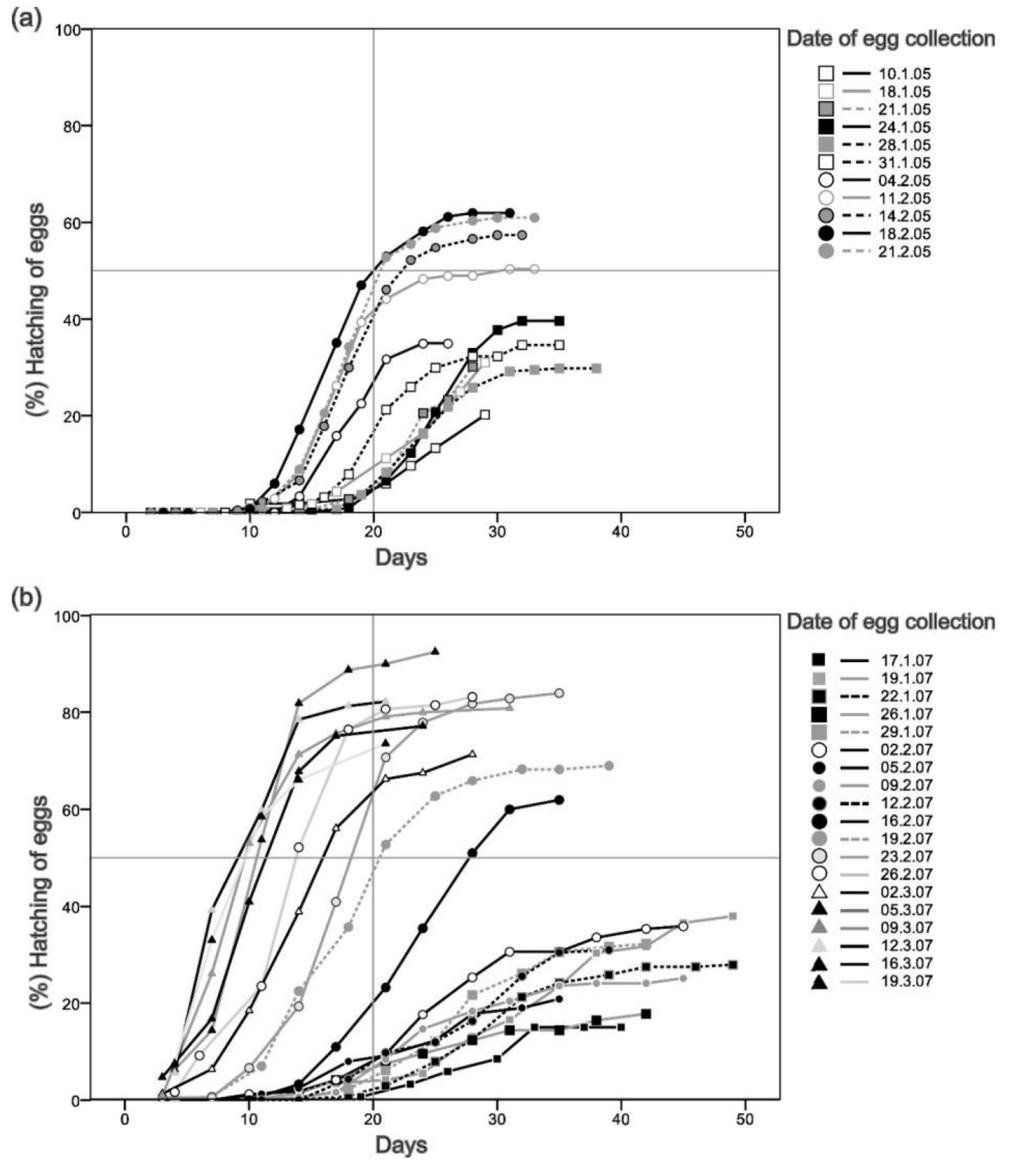
Hatching progression with time for eggs of the European red mite collected at different dates and maintained in controlled conditions at 20 ºC for the years of (**a**) 2005 and (**b**) 2007. The date for diapause termination is set at the earliest date that reaches 50% of egg hatching in 20 days of incubation at 20 ºC

Figure 3 shows the progression of egg hatching with time depending on the date at which egg masses were collected and exposed to controlled laboratory conditions. The first date at which at least 50% of eggs hatched after a period of 20 days at controlled conditions was the 18th Feb for 2005 and the 23th Feb for 2007, which were established as dates for diapause termination.

### Temperature threshold and degree-days required for postdiapause development

**Fig. 4:**
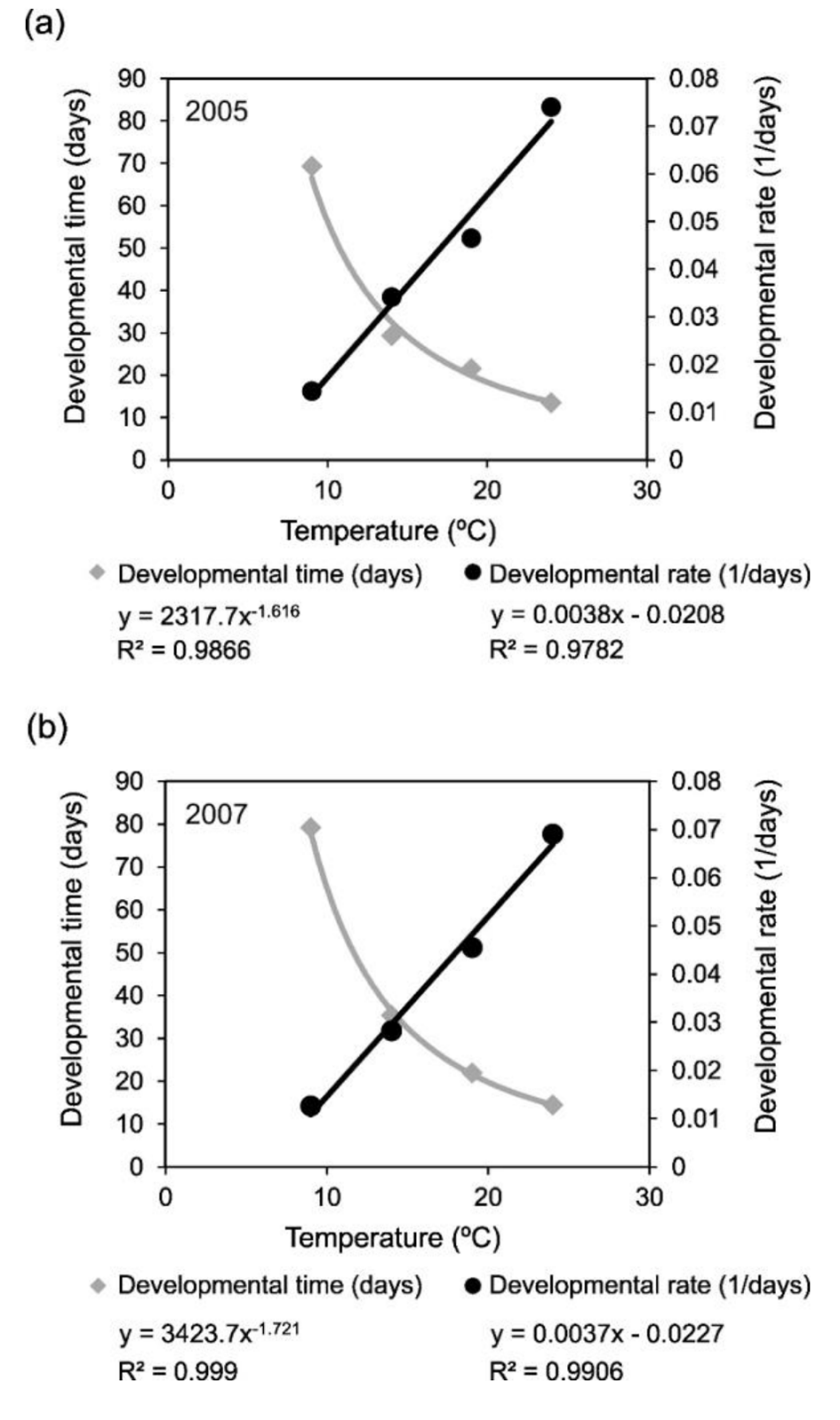
Temperature threshold for diapause termination and degree-days required for postdiapause development in the European red mite for the years of (**a**) 2005 and (**b**) 2007

Figure 4 shows the developmental rate at different temperatures for those egg masses collected after the date of diapause termination. The equation that emerged from linear regression allowed for the calculation of the temperature threshold and the degree-days required for postdiapause development. The temperature thresholds based on the adjusted regression lines (2005: y = 0.0038x-0.0208; 2007: y = 0.0037x-0.0227) were established at 5.47 ºC and 6.13 ºC for 2005 and 2007, respectively. The degree-days required for postdiapause development were established at 263.2 and 270.3 for 2005 and 2007, respectively. Based on these temperature threshold and degree-days estimated for postdiapause development, egg hatching of 50% of the population was estimated on the 26th of April in 2005 and on the 25th of April in 2007.

## Discussion

Diapause breaking in leaf-feeding herbivores is often synchronized with that of their deciduous tree hosts, given that food availability is a key factor to nourish the first generation of overwintering organisms (Van Asch and Visser 2007). The importance to accurately predict diapause has implications for a proper management of the pest population, for example to adjust the timing or frequency of pesticide treatments (Luedeling et al. 2011) and to estimate the biocontrol potential of natural enemies (Hardman et al. 2013). In this work, we expand the knowledge on the abiotic factors that influence diapause termination of the European red mite *Panonychus ulmi*. Our study over three years describes the temperature and photoperiod requirements necessary for breaking the diapause in the mite populations collected in northern Spain. Furthermore, we provide additional information on the temperature threshold and degree-days required for postdiapause development. Altogether, this will allow to further improve modelling approaches to accurately predict and describe the population dynamics of this mite species, an important pest on many fruit crops such as apple, pear, walnut and grapevine.

The physiological process of diapause has been examined for at least four species within the genus *Panonychus* (Lees 1953; Gotoh et al. 1994). Diapause occurs at the late blastula stage, a process that for *P. ulmi* takes approximately 30 h after egg deposition at 24–25 ºC under continuous light conditions. Once winter eggs enter diapause, they undergo through a period of latency until favorable environmental conditions signal diapause termination. Koveos and Broufas (1999) showed that eggs collected from the field early on the season (i.e. before exposure to cold temperatures) required a longer period for diapause breaking at 20 ºC than late collections. Our study confirms that changing the duration the winter eggs experience at a specific cold temperature can have a significant impact not only in the time required for diapause termination but also in hatching success.

Our findings suggest a positive relationship between the length of the period of exposure to cold temperatures and hatching success. This result is coincident with that of Cranham (1971) who showed that increasing the period of exposure to cold from 60 to 200 days reduced the time required for *P. ulmi* diapause termination and resulted on a higher hatching success. In our study, exposure to only 10 days of cold was enough to report some degree of egg hatching. This period was shorter than previously reported (Lees 1953; Koveos and Broufas 1999) that established a minimum of 40–50 days before diapause termination. These results may be explained by the different moment of collection of the field populations used for the experiments because *P. ulmi* females can extend their period of oviposition over 6–8 weeks (Cranham 1973). This may translate into a different accumulation of temperature on overwintering eggs from early and late oviposition events; thereby influencing all parameters under study, and confounding comparisons between locations and studies.

The specific temperature that the eggs experienced during diapause also modulated the process and impacted on the percentage of eggs that hatched. Our results showed the highest egg hatching rates upon exposure of eggs to a temperature of 4–8 ºC. This result is consistent with previous studies reporting that the most efficient diapause breaking temperature for *P. ulmi* were in the range of 1 to 9 ºC (Lees 1953) and 0 to 5 ºC (Cranham 1971, 1972). These authors also studied the minimum and maximum temperature at which diapause could be terminated, but there was not consensus at the minimum temperature at which eggs could develop. For example, Cranham (1972) showed egg hatching at -3 ºC and − 6 ºC; while Lees (1953) reported that no emergence occurred at -5 ºC. Cranham (1973) further attributed these results to intraspecific variation and showed that six different populations collected at different sampling points in the southeast of England showed variable responses to temperature in relation to diapause termination. These results correlated well with field observations because the populations that terminated diapause the earliest in the field, were also breaking diapause earlier at the fixed temperature of 0 ºC under laboratory conditions. García-Marí et al. (1991) also reported variation between field populations, establishing a range of 90–100 days required for egg hatching upon exposure to 1 ºC; depending on the area of collection from the field.

Earlier work reported that diapause breaking by *P. ulmi* eggs after a sufficient period of exposure to cold can occur between 9 and 25 days at high temperature (Lees 1953). In natural conditions, this translates to 150–200 degree-days required prior to diapause termination. Light et al. (1968) indicated that eggs require 100 to 150 days at 5 ºC to complete the diapause. García-Marí et al. (1991) showed that hatching can occur at 10–12 days after sufficient exposure to cold temperatures. Our results are in agreement with the previously reported because eggs exposed to 100 days of temperature at a range of (0–8 ºC) hatched in a period of 10–20 days.

Most studies evaluated the influence of temperature and the duration of cold days as the main factors that determine diapause termination. However, the impact of photoperiod on diapause breaking is still debatable. Lees (1953) claimed that photoperiod was not an important parameter for diapause termination because eggs hatched when maintained at 25 ºC in total darkness. Hueck (1951) speculated that light was a stimuli for hatching, as fewer individuals emerged during the night and larva showed a strong phototaxy (i.e. movement towards light) on the experimental arena. Our results showed that photoperiod is a significant factor for diapause termination because the percentage of hatched eggs increased by extending the period of exposure to light conditions. In other species that also overwinter at the egg stage, such as *Labops hesperius* Uhler (Hemiptera: Miridae), photoperiod was also a factor in egg hatching (Fuxa and Kamm 1976). Similarly, photoperiod regulated diapause in *Tetranychus urticae* (Koveos and Veerman 1996), although this comparison should be taken with caution, as *T. urticae* experiences diapause at the adult stage. Although the natural photoperiod is dependent on latitude and therefore predictable all year round, these results may be relevant for cases in which dormant plant material is moved to other areas of the world, potentially carrying winter eggs. Proportional precautionary measures should be put in place given that an increased photoperiod can stimulate diapause breaking for those eggs with short exposure to cold temperatures.

Variability on T_50%_ between years may be influenced by the meteorological variation experienced by egg masses prior to collection from the field. Koveos and Broufas (1999) collected eggs from a field site at different sampling times (early and late in winter season) and exposed them to a short or long photoperiod to evaluate diapause termination at the stable temperature of 20 ºC. As stated above, eggs collected at the end of the season had experienced a longer period of time at cold temperature and this reduced the T_50%_ from 60 to 20 days, approximately (Koveos and Broufas 1999). A similar trend was observed by Cranham (1971) and T_50%_ could differ up to 3 weeks depending on the location and time of collection. Our results also showed a significant effect of the period of time exposing the mites to cold temperature on T_50%_. Other environmental factors such as temperature or photoperiod may also influence T_50%_ but to a lower extent. Koveos and Broufas (1999) report minor effects of photoperiod on T_50%,_ however, a significant interaction was found between sampling date and photoperiod at which eggs were allowed to develop. Although the effect might be minor, exposure to a long photoperiod decreased the time required to break diapause on those eggs collected earlier in the season, but not at the end of the season. This is in agreement with our results, because although we did report an effect of photoperiod on emergence rate, we did not observe a consistent effect on T_50%_ for 2004 and 2005. Overall, our results and those of previous studies highlight the importance to adjust modelling of diapause termination to the specific meteorological conditions of each year and location of study, and emphasize the interest of obtaining data on local mite populations.

Using field collected egg masses, the date of diapause termination was established at the third week of February, which was consistent with earlier studies conducted in the Mediterranean area (Broufas and Koveos 2000). Likewise, postdiapause development was studied under laboratory conditions obtaining values of temperature threshold ranging between 5 and 6 ºC and 263–270 degree-days above the temperature threshold. These range of values are within the values previously reported for *P. ulmi* that established the temperature threshold between 5 and 7 ºC (Lees 1953; Cranham 1971, 1972; Herbert and McRae 1982). Only Bostanian et al. (2007) obtained a temperature threshold of 10.1 ºC using data collected by Herbert (1981); which is a value above the rest of the studies. This may likely be due to differences in methodological approaches as the temperature range for Herbert (1981) was based on data from (15–21 ºC) while in the other studies such that of Herbert and McRae (1982), a broader range was applied (8–20 ºC) allowing for a more precise estimation of the temperature threshold. Our estimation of temperature thresholds and degree-days required for postdiapause termination predicted a 50% hatching of the winter eggs by the last week of April. At this time point, the phenology of apple is over the full bloom stage, therefore, the availability of leaf material for *P. ulmi* would be ensured.

Under a climate change scenario of increasing temperatures, the number of the European red mite generations in a year is expected to increase (Luedeling et. 2011). To accurately predict population dynamics, modelling could benefit from adding additional parameters that take into account diapause as it was previously developed for the codling moth (*Cydia pomonella* L.) (Pak et al. 2022). Additionally, modelling parameters can be customized for each specific region of the world. For example, physiologycal models on *P. ulmi* such as the one used by Luedeling et al. (2011), require to establish parameters such as the threshold for minimum temperature for development and also, the date in which egg hatching is first observed in the season. Our results indicate that the minimum developmental temperature threshold for the European red mite was five degrees Celsius lower in our region of study than that reported in Luedeling et al. (2011). These significant differences may influence the outcome of physiological models and call for studies at a local scale that could improve the regional prediction of mite population dynamics.

## Conclusions

In this study, we collected *P. ulmi* overwintering eggs from the field and exposed them to several temperatures and photoperiods to determine the parameters relevant for diapause breaking. We report that a period of cold is required for maximum egg hatching, which is modulated by temperature and photoperiod. This modulation of diapause termination may allow mites to adapt to local conditions and synchronize their populations with the availability of foliage; a key requirement to nourish first generation. Mistiming egg hatching may otherwise have strong fitness penalties for herbivores feeding on deciduous trees and that cannot survive starvation for a long period (Van Asch and Visser 2007). We add information to the specific conditions of diapause breaking in northern Spain, at a location under temperate/Mediterranean climate. The information provided (i.e. egg hatching rate, T_50%,_ temperature threshold, degree-days for postdiapause development) can be used in modelling approaches to improve the prediction capability, for example, to estimate the hatching success of larvae from winter eggs. Ultimately, this knowledge could contribute to optimize pest control strategies of the European red mite in fruit orchards.

## Data Availability

No datasets were generated or analysed during the current study.
